# Therapeutic potential of interleukin-21 in cancer

**DOI:** 10.3389/fimmu.2024.1369743

**Published:** 2024-04-04

**Authors:** Gheorghita Isvoranu, Marioara Chiritoiu-Butnaru

**Affiliations:** ^1^ Department of Animal Husbandry,” Victor Babeș” National Institute of Pathology, Bucharest, Romania; ^2^ Department of Molecular and Cell Biology, Institute of Biochemistry of the Romanian Academy, Bucharest, Romania

**Keywords:** IL-21, NK cells, T cells, cancer immunotherapy, engineered cytokines, checkpoint inhibitors, adoptive cell therapy, oncolytic vaccinia virus

## Abstract

Interleukin-21 (IL-21) is an immunostimulatory cytokine which belongs to the common gamma-chain family of cytokines. It plays an import role in the development, differentiation, proliferation, and activation of immune cells, in particular T and natural killer (NK) cells. Since its discovery in 2000, IL-21 has been shown to regulate both adaptive and immune responses associates with key role in antiviral and antitumor responses. Recent advances indicate IL-21 as a promising target for cancer treatment and encouraging results were obtained in preclinical studies which investigated the potency of IL-21 alone or in combination with other therapies, including monoclonal antibodies, checkpoint inhibitory molecules, oncolytic virotherapy, and adoptive cell transfer. Furthermore, IL-21 showed antitumor effects in the treatment of patients with advanced cancer, with minimal side effects in several clinical trials. In the present review, we will outline the recent progress in IL-21 research, highlighting the potential of IL-21 based therapy as single agent or in combination with other drugs to enhance cancer treatment efficiency.

## 1 Introduction

Cancer is a major challenge of modern medicine and significant resources have been allocated to develop more efficient and safer therapies. In this context, immunotherapy, which has higher specificity and shows reduced side effects compared with conventional therapies has become a sustainable cancer treatment in the last years. The aim of immunotherapy is to drive the patient’s self-immune system into fighting the tumor, designed to amplify or suppress an immune response. Cytokines are key mediators of the immune system, with crucial role in regulating the immune response; in this context exploiting the antineoplastic capacity of cytokines to design efficient therapies has increased.

**Figure 1:**
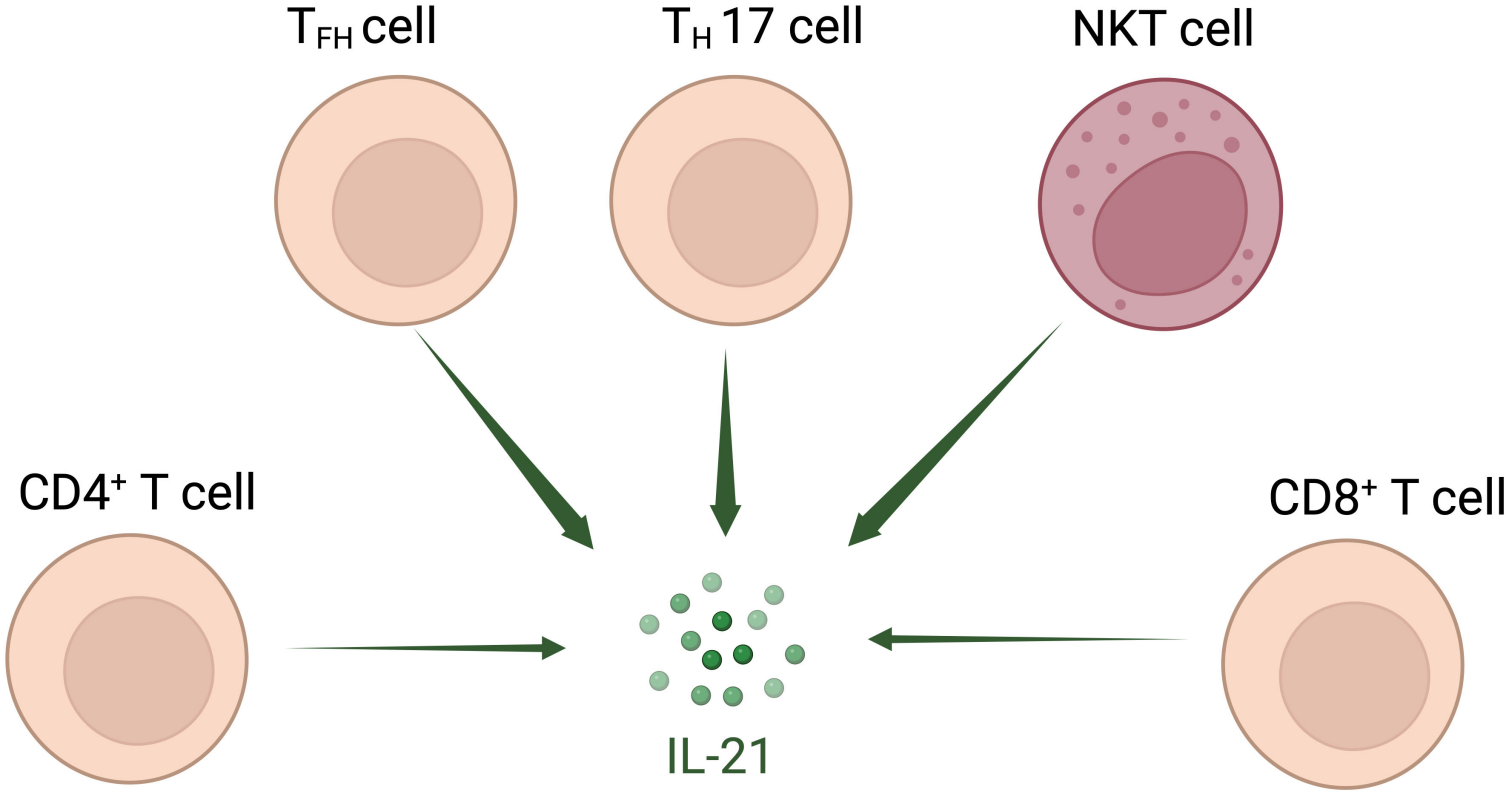
Interleukin-21-secreting cell types. IL-21 is secreted mostly by T follicular helper (T_FH_) and T_H_17 cells and Natural Killer T (NKT) cells; other CD4^+^ and CD8^+^ T cells also secrete IL-21 at lower level.

**Figure 2:**
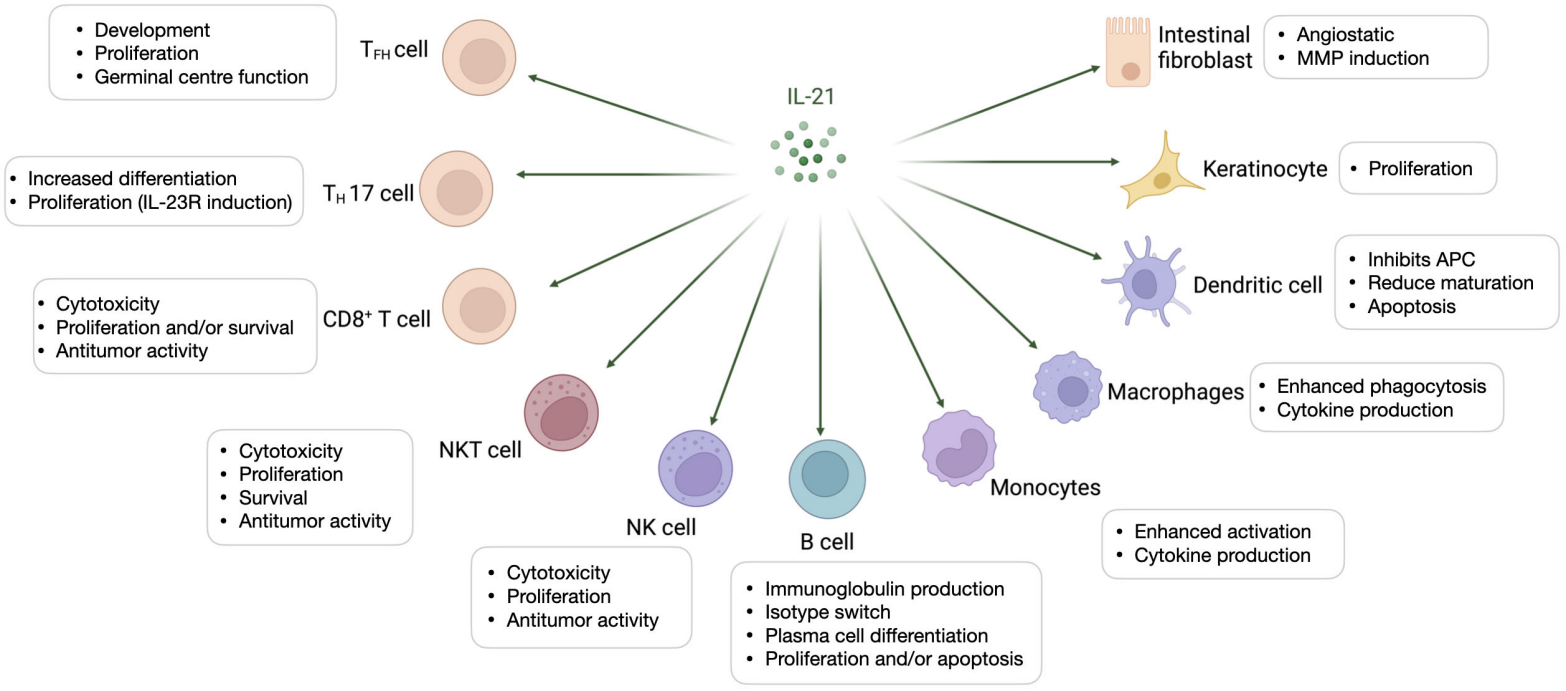
Interleukin-21-targeted immune cells. IL-21 modulates proliferation, maturation, and activation of several cell types amongst which T cells, Natural Killer T (NKT) cells, Natural Killer (NK) cells, B cells, monocytes, macrophages, and dendritic cells (DCs), keratinocytes, and intestinal fibroblasts.

Interleukin (IL)-21 is an immunostimulatory cytokine belonging to the common gamma-chain family of cytokines which plays an important role in immune cells activation and proliferation, in particular T and NK cells. Therefore, IL-21 has been proposed as a good candidate for cancer treatment, either alone or in combination with other therapeutic agents. In 2000 Ozaki et al. discovered a new type I cytokine receptor which was named Novel Orphan Interleukin Receptor (NILR) (1). Soon after, the natural ligand for NILR was identified by functional cloning: a cytokine structurally related to the cytokine family with four alpha helix bundle structure. This new cytokine was named interleukin 21, and NILR was renamed the IL-21 receptor (IL-21R) (2). IL-21 is produced mostly by CD4^+^ T cell subsets (primarily T follicular helper (T_FH_) and T_H_17 cells) (2, 3) and Natural Killer T (NKT) cells (4) with lower level being produced by other lymphohematopoietic cells (**Figure 1**). It has a pleiotropic effect on a wide range of immune cells including T cells, NKT cells, NK cells, B cells, monocytes, macrophages, and dendritic cells (DCs), keratinocytes, and intestinal fibroblasts as summarized in **Figure 2** (5–7). The human IL-21 gene is located on chromosome 4q27 and 180kb apart from IL-2 gene, while in mice the gene is located on chromosome 3 and separated by 95 kb from IL-2 gene (8). Although the two genes present similar intron and exon structures, they are differentially regulated. In humans, the gene encoding for IL-21 receptor is located on chromosome 16, immediately downstream of the α receptor for IL-4 and shows great sequence homology with the β receptor for IL-2. IL-21 and IL-21R in humans and mice show over 60% sequence homology, with significant conservation of cytokine-receptor interaction regions (2).

IL-21 signals through a receptor complex consisting of IL-21R and the common cytokine receptor γ chain (γc, CD132) (9), which makes IL-21 a member of the common γ-chain cytokine family along with IL-2, IL-4, IL-7, IL-9, IL-15. IL-21R is expressed on immune cells in both lineages, lymphoid and myeloid, as well as non-immune cells, such as keratinocytes and epithelial cells (10–12). The presence of IL-21R was also reported in many hematological malignancies (13).

The dimerization of IL-21R and γc leads to the recruitment and phosphorylation of Janus-Activated Kinase (JAK)1 and JAK3, which subsequently phosphorylate and activate Signal Transducer and Activator of Transcription (STAT)3, STAT1, and at a lower level STAT5. IL-21 can also activate the MAPK (Mitogen activated protein kinase) and PI-3K-AKT (Phosphatidylinositol-3-kinase-AKT) signaling pathways (14). It preferentially activates STAT3, which is critical for its effects on B-cell and T-cell differentiation. IL-21 stimulation also leads to the activation of STAT1, at least in T cells, B cells, and conventional dendritic cells (cDCs) whereas IL-2 and IL-15 activates STAT5A and STAT5B.

IL-21 signaling via its receptor induces expression of genes associated with activation of innate immunity and Th1 response such as granzymes A and B, cyclins, interferon-γ (IFN-γ), T-bet, IL-2Rα, IL-12Rβ2, IL-18R, and myeloid differentiation factor 88 (MyD88) (15–18). It promotes the generation of CD8^+^ memory stem T (T_SCM_) by upregulating expression of *T-box 21* (*TBX21*) and suppressor of cytokine signaling 1 (*SOCS1*) (19). Also, IL-21 mediates B cell survival and differentiation by regulating *Prdm1* (which encodes for B lymphocyte-induced maturation protein-1 BLIMP1), *Bcl6* and *Bim* genes (20, 21).

### 1.1 Costimulatory Actions of Interleukin-21 on T and NK cells

IL-21 has a crucial role in development, differentiation, survival, and functionality of lymphocytes, promoting the antitumor response of both adaptative and innate immune cells.

*CD8^+^ T cells*: Many studies indicated that IL-21 plays an important role in CD8^+^ T cell survival and memory formation (22, 23). IL-21 along with IL-7 and IL-15 regulate expansion, survival and effector function of both memory and naive T cells (24). Moreover the tumor-specific CD8^+^ T cells induced by IL-21 stimulation exhibited better antitumor effects compared with T cells generated by exposure to IL-2 and IL-15 (25). Stimulation of naive T cells with IL-21 induced differentiation of memory stem-like T cells that display a central memory phenotype, mediating a higher and more robust antitumor activity *in vitro* and *in vivo* (26–28).

*CD4^+^ T cells*: Functional plasticity and the ability to differentiate into various subsets allow CD4^+^ helper T cells to continuously adjust the immune responses to multiple pathological situations. IL-21 was shown to impair the development and homeostasis of regulatory T-cells (T_reg_), which are abundant in tumor tissues, both in human and mice (29, 30). The presence of T_h_17 cells and T_reg_ cells was reported in various tumors and role of these cells in antitumor immunity depends on the tumor type (31). Liu et al. indicated that the IL-17-producing cells could promote angiogenesis and development of colorectal carcinoma (32), while Muranski et al. found that tumor-specific T_h_17 cells mediated highly efficient antitumor effect in an established B16 melanoma model (33). In this context, it was shown that IL-21 is necessary for T_h_17 differentiation (34–37). T_h_9 cells are another subset of CD4^+^ T cells, demonstrated potent antitumor effects in solid tumors (38–40). IL-21 was shown to inhibit the formation of T_h_9 cells due to induction of B cell lymphoma 6 (BCL-6) transcriptional factor of these cells. It enhanced the expression of BCL6 that binds to the IL-9 promoter, competing with STAT5 in these cells (41).

*NK cells:* IL-21 favors differentiation and functional activation of NK cells, including improvement of NK cell-mediated antibody-dependent cellular cytotoxicity (ADCC). IL-21 along with IL-2 or IL-15 induced the phenotypic maturation and potent functional activation of murine NK cells in a NKG2D and perforin dependent manner (42). IL-21 alone was not able to support the NK cell viability and decreased IL-15-mediated expansion of resting NK cells (43). Our group showed that IL-21 alone had a low effect on of murine NK cells proliferation without survival benefits, while IL-21 in combination with IL-15 induced proliferation, enhanced survival and functional activation of mouse NK cells (44). Similarly, studies using human cells showed that IL-21 stimulates proliferation and maturation of NK cells isolated from bone marrow or cord blood cultured in presence of IL-15, Flt3-L (Fms-like tyrosine kinase receptor 3 ligand) and SCF (stem cell factor) (2, 45). Peripheral blood mononuclear cells (PBMCs) grown in medium supplemented with IL-21 displayed an increased proliferation of CD56^bright^ NK cells and augmented antitumor activity against K562 cells of CD56^dim^ NK cells, without interferon-γ and tumor necrosis factor-α (TNF-α) production (46). Nonetheless, IL-21 did not increase NK cells proliferation in healthy and Human Immunodeficiency Virus (HIV)-infected donors, but increased the perforin production and enhanced NK cytotoxicity against K562 target cells (47). Treatment with IL-21 induced NK cell ADCC and high production of IFN-γ in response to cetuximab-coated pancreatic tumor cells (48). Exposing NK cells to a combination of IL-21 and IL-15 does not have major effects on cells expansion, but their antitumor effect is considerably increased (49). Despite its capacity to enhance NK cell cytotoxicity IL-21 reduced the life span of NK cells by markedly triggering apoptosis (50). Similar to T cells, IL-21 can induce the expansion of “memory-like” NK cells, data documented in a mouse model of tuberculosis (51).

## 2 Interleukin-21 exploitation in preclinical studies

**Figure 3:**
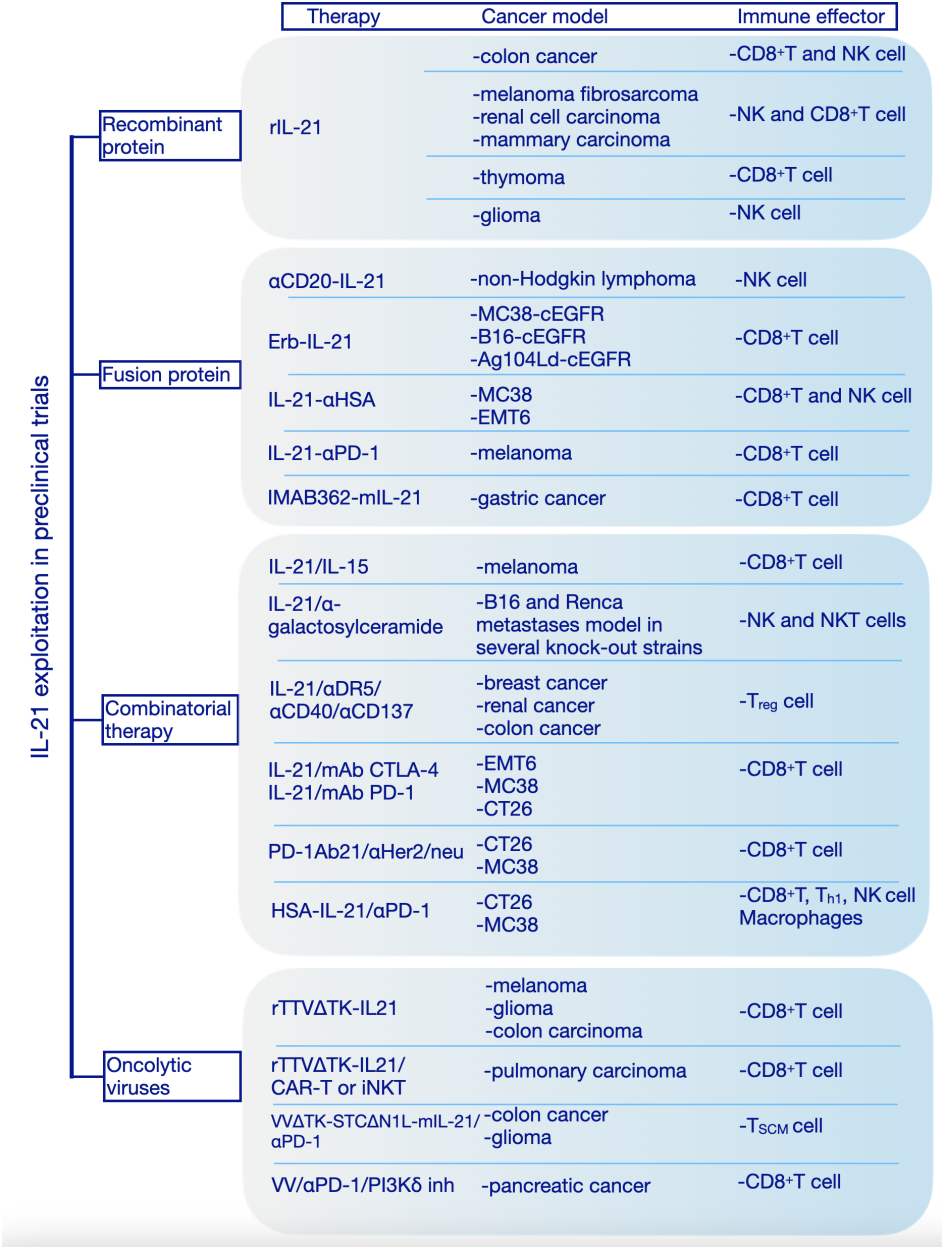
Interleukin-21 exploitation in preclinical studies. We have categorized the preclinical studies in 4 major categories based on the therapeutic approach: recombinant protein, fusion protein, combinatorial therapy, oncolytic viruses. rIL-21, recombinant interleukin-21; IL-15, interleukin-15; mAb, Monoclonal antibodies; CTLA-4, Cytotoxic T-lymphocyte associated protein 4; PD-1, Programmed cell death protein 1; HSA, human serum albumin; cEGFR, Common sensitizing mutations in epidermal growth factor receptor; Erb-IL-21, Erbitux-based IL-21 tumor-targeting fusion protein; IMAB362, Zolbetuximab; mIL-21; mouse IL-21; Her2/Neu, human epidermal growth factor receptor-2 receptor; CAR, chimeric antigen receptor; TTVΔTK, vaccinia virus Tian Tan strain with deletion of thymidine kinase gene; VVΔTK-STCΔN1L, vaccinia virus Lister strain with deletion of thymidine kinase and virulence factor N1L genes; VV, vaccinia virus; PI3Kδ inh, phosphoinositide 3-kinase-δ inhibitor; MC38, murine colon adenocarcinoma cell line; B16, murine melanoma cell line; Ag104Ld, mouse fibrosarcoma cell line; EMT6, murine mammary carcinoma cell line; CT26, murine colorectal carcinoma cell line; NK cell, Natural Killer cell; T_h_1, T helper 1; T_reg_, T regulatory; T_SCM_, stem cell-like memory T cell; iNKT, Invariant Natural Killer T cell.

IL-21 receptor is expressed on vascular endothelial cells (ECs) and this might explain the possible role of IL-21 on inhibition of tumor angiogenesis which has been addressed by both *in vitro* and *in vivo* studies (52). The ability of IL-21 to regulate diverse immune cells together with its angiostatic properties confer this cytokine a potent ability to drive tumor cells clearance. Many studies have shown that IL-21 can augment the antitumor response mediated by CD8^+^ T cells, NK cells and B cells (**Figure 3**).

The first evidence of IL-21 antitumor activity *in vivo* was shown in a murine model, when colon carcinoma cells transduced to express IL-21 were rejected, the antitumor effects being dependent on T and NK cells, in this case (53). Also, the administration of plasmids expressing IL-21 exhibits significant antitumor activity, mediated by NK cell and T cells, against melanoma fibrosarcoma (54), renal cell carcinoma and mammary carcinoma (42) subcutaneous head and neck squamous cell carcinoma (55). In a thymoma model expressing ovalbumin (OVA) intraperitoneal (i.p.) administration of recombinant IL-21 significantly inhibited tumor growth and induced durable survival compared to IL-2 and IL-15, the effect being CD8^+^ T cell-dependent, IL-21 increasing the number and cytotoxicity of (OVA) CD8^+^ T cells (25). Although, both routes of administration of IL-21, subcutaneous (s.c.) or i.p., induced antitumor effects in mice bearing established melanoma or renal cell carcinoma, only s.c. route inhibited the growth of large tumors. This effect was dependent on CD8^+^ T cells and IL-21 increased the density of tumor infiltrating CD8^+^ T cells (56). Also, it was shown that IL-21 could induce NK cell-mediated rejection of tumors, independently of the adaptive immune response (57). In a mouse model of intracranially implanted gliomas, administration of IL-21 by stereotactic injections showed significant tumor rejection. In this case, IL-21 especially influenced the NK cells and antibody-mediated immunity (58).

Because recombinant IL-21, although promising, provided modest protection against cancer, in recent years new approaches, based on cytokine engineering, are rapidly developing to improve half-life, tumor targeting and reduce side effects. The bifunctional protein, αCD20-IL-21 fusokine, obtained by fusion IL-21 to anti-CD20 antibody Rituximab, demonstrated superior anti-cancer potential compared to its individual components. In addition to stabilizing and extending the half-life of IL-21, the αCD20-IL-21 enhanced IL-21R-mediated signaling which results in direct lymphoma cytotoxicity and increased NK cell-mediated cytotoxicity (59).

Deng et al. generated an Erbitux-based IL-21 fusion protein (Erb-IL-21) to target tumor cells expressing chimeric EGFR (cEGFR). This fusion protein exhibited extended half-live and improved antitumor effects. Also, Erb-IL-21 presented much lower toxicity than Erb-IL-2, even at high doses and intraperitoneal treatment with Erb-IL-21 limited the tumor growth in well-established tumor models (MC38-cEGFR, B16-cEGFR, and Ag104Ld-cEGFR). Here, tumor control depended on CD8^+^ T cells, Erb-IL-21 selectively expanded existing intratumoral PD-1^int^Tim-3- CD8^+^ functional T cells. Combining Erb-IL-21 with anti-PD-L1(Programmed Cell Death Ligand 1) antibody as treatment for mice bearing tumors dramatically reduced the tumor volume and enhanced survival, whereas the administration of Erb-IL-21 with anti-CTLA-4 (Cytotoxic T-lymphocyte-associated protein 4) induced the tumor clearance in all mice (60).

To further increase the stability and prolong its half-life, the C-terminus of recombinant human IL-21(rhIL-21) was fused with a nanobody targeting human serum albumin (HSA) to generate a new product, IL-21-αHSA. IL-21-αHSA showed an enhanced antitumor effect when was administrated either alone or in combination with PD-1 and TIGIT (T cell immunoreceptor with Ig and ITIM domains) blockades (61).

Using another approach, by fusion of highly attenuated IL-21 mutein variant (R9E:R76A) to the C-terminus of the anti-PD-1 antibody, a bifunctional protein was obtained which provided protection in a human melanoma mouse model resistant to anti-PD-1 therapy. This fusion protein can block PD-1/PD-L1 interactions while presenting IL-21 to PD-1^+^ cells (62). In 2020 a clinical trial was designed to evaluate the safety, tolerability and estimated dosing of this bifunctional protein (named AMG 256 or Latikafusp) in patients with advanced solid tumors (63, 64) (NCT04362748).

Another construct which combined tumor-targeting and effective activation of the local antitumor immune response is anti-Claudin18.2-IL-21 fusion protein (IMAB362-mIL-21). IL-21 was fused to the monoclonal antibody IMAB362 (Zolbetuximab) that binds to Claudin18.2, a target antigen specific to cancer cells. IMAB362-mIL-21 proved potent antitumor effects and increased safety both *in vitro* and *in vivo* (65). Overall, combining IL-21 with other immuno-stimulants, monoclonal antibodies that recognize specific tumor antigens or chemotherapy for cancer treatment showed better performances than monotherapy (66).

It is widely accepted that IL-21 synergistically acts with other cytokines to promote an antitumor response. Therefore, it was shown that IL-21 in combination with IL-15 enhanced the antitumor effect of CD8^+^ T lymphocytes leading to tumor regression in established melanoma (24). Combinations of IL-21 with α-galactosylceramide conferred higher antitumor activity in experimental tumor metastases by activation of NK cells induced by NKT cells (57). Moreover, another study demonstrated IL-21 enhanced the efficiency of a triple monoclonal antibody (anti-DR5/anti-CD40/anti-CD137) to eradicate established tumors (67).

Another approach to enhance antitumor activity is to combine IL-21 with checkpoint receptors blockade, obtaining thus an increased efficiency compared to single agents. Within this context, Lewis et al. tested the effects of IL-21 in combination with monoclonal antibodies (mAbs) for CTLA-4 and PD-1 regulatory receptors after tumor establishment in several mouse models. In an EMT-6 mammary carcinoma model IL-21 and mAb CTLA-4 combination therapy induced complete regression in half of the mice and noticeable delays in tumor growth for the rest of mice employed in the study, while the combination of IL-21 and mAb PD-1 displayed no noticeable effects. However, for a B16-F10 subcutaneous melanoma model combinatorial therapy of IL-21 and CTLA-4 or PD-1 resulted in smaller tumors without complete regression in any of the mice used for experiments. Also, in intravenous B16-F10 tumor model, combination of IL-21 with each of the two antibodies targeting the regulatory receptors induced fewer lung metastases. In the MC38 colon carcinoma model combined therapy was effective, both combinations inducing complete regression in more than 50% of mice. In contrast, using a CT26 colon carcinoma model to evaluate the combinatorial therapy, only IL-21 and mAb CTLA-4 combination showed efficient results, five of eight mice presented complete regression of tumor with the IL-21 and mAb PD-1 combination having no antitumor effects in this model. Treatment of colon carcinoma tumors with either of these two combinations induced enhancement of CD8^+^ T cells infiltration into tumors, increased effector memory T cells, and decreased central memory T cells (68).

Aiming to overcome this restriction, Li et al. adopted an alternative strategy and generated a PD-1Ab21 construct by fusing an anti-PD-1 antibody with IL-21 to increase the generation of memory stem T cells and induce the expansion of tumor-specific CD8^+^ T cells with a memory phenotype. In CT26 and MC38 tumor models the PD-1Ab21 intraperitoneal administration revealed robust antitumoral effects compared to nontreated group and groups treated with anti-PD-1 antibody or mAb PD-1 plus IL-21 groups. The potent antitumor effect of PD-1Ab21 was also highlighted in combination with anti-Her2/neu antibody or with tumor antigen vaccination. In a TUBO tumor model generated by inoculation of tumor cells that overexpress the HER2 (neu) oncogene, the treatment with antiHer2/neu antibody combined with PD-1Ab21 significantly inhibited the growth of tumors. Treatment with PD-1Ab2 after intradermal injection of poly I:C and OVA peptide vaccine delayed tumor growth in B16-OVA-bearing mice (69).

Similarly, attempting to prolong the half-live and enhance the antitumor activity of IL-21 a new product was created by fusing an anti-HSA nanoantibody with IL-21. Combination of HSA-IL-21 fusion protein with PD-1 blockade significantly reduced tumor growth in an established MC38 tumor model. The effects of this therapeutic combination were observed on immune cells from the TME and peripheral lymphoid organs. The mechanism of action of this combined therapy seems to rely on the increased expression of IL-21R on immune cells in the TME that drive TILs into a hyperactivated state controlled multiple immune checkpoints, such as Tim-3, Lag-3 (Lymphocyte-activation gene 3), and CD39. Also, the HSA-IL-21 and PD-1 blockade combination increased the dendritic cells type 1 and M1 macrophages. The antitumor efficacy of HSA-IL-21 and PD-1 was greatly enhanced by triple or quadruple combinations with Tim-3 or Lag-3, without apparent toxicity (70).

Cytokine-armed oncolytic viruses have been extensively studied in pre-clinical tumor models as a new treatment for cancer. Targeted delivery of cytokines into the tumor microenvironment can potentially reverse “cold” tumors into “hot” tumors with high lymphoid cells infiltration. An *in vitro* study reported an oncolytic adenovirus co-expressing chemokine CCL21 and IL-21 that could selectively replicate in TERTp-positive tumor cells (Ad-CCL21-IL-21 virus) was able to induce direct lysis of tumor cells by cytotoxic T cells (71). Oncolytic viruses armed with interleukin-21 demonstrated superior antitumor efficacy in several cancer models. In three mouse tumor models (melanoma, glioma, colon carcinoma) Chen at al. showed that intratumorally administration of an IL-21-armed recombinant vaccinia virus, rTTVΔTK-IL-21, led to selective enrichment of immune effector cells at tumor site, long-lasting tumor regression and improved survival. Also, rTTVΔTK-IL-21 showed synergic actions with CAR T therapy or invariant nature killer cell (iNKT) for cancer immunotherapy in a humanized mouse model of pulmonary carcinoma (72). Moreover, treatment with a vaccinia virus armed with IL-21 effectively controlled tumor growth and improved survival for colorectal cancer model mice (73). In a glioma GL261 model vaccinia virus expressing IL-21, VVΔTK-STCΔN1L-mIL-21, in combination with anti-PD1 antibody inhibited tumor growth leading to long-term survival for 80% of treated mice. After treatment, the stem cell memory T cells in the spleen and the expression of PD-L1 in the tumor were significantly increased, suggesting that this combination could be an efficient therapeutic option for patients with glioblastoma (74). Combining a vaccinia virus expressing IL-21 treatment with anti-PD1 and a PI3Kδ (phosphoinositide 3-kinase-δ) inhibitor to prevent virus uptake by macrophages was able to significantly inhibit tumor growth and increased overall survival in an established pancreatic tumor model. This treatment induced a significant increase in systemic T cell response, with the presence of effector CD8^+^ T cells being considerably increased in the blood, spleen and tumor (75).

## 3 Interleukin-21-candidate for adoptive cell therapies

Adoptive cell therapy (ACT) or cellular immunotherapy involves the transfer of immune cells to a patient to eliminate tumor cells. For this, the immune cells which are collected from the patient itself or another individual (donor), are expanded *ex vivo* or/and genetically engineered to enhance their antitumor abilities. ACT was usually designed using T cells due their capacity to bind specific antigens on the surface of tumor cells, which ensures targeting specificity. However, more recently, other immune cells such as NK cells, macrophage, γδT cells, and NKT cells begun to be used in cancer treatment. In contrast to the major advantages of ACT, using immune cells for cancer therapy also presents considerable challenges such as insufficient numbers of cells, which need to be activated and maintain their active state long enough to kill tumor cells, intra-tumoral suppression of cell functions, and toxicity issues therefore limiting the implementation of this therapeutic approach on larger scale.

### 3.1 T cells-based adoptive cell therapy

T cells-based ACT set the ground for implementation of this curative approach which started to gain interest roughly four decades ago. In this regard, several studies have investigated the potential of harnessing T cell based ACT involving IL-21 to improve cancer treatment and we have highlighted some of them below (76, 77).

Tumor infiltrating lymphocytes (TILs) cultivated in the presence of K562 artificial antigen-presenting cells engineered to secrete IL-21 exhibited a less-differentiated young phenotype and presented superior antitumor activity upon adoptive transfer, without collateral expansion of T_regs_ (78). Additionally, IL-21 in combination with IL-2 and IL-15 was used to obtain the central memory tumor infiltrating T-cells from patients with glioblastoma and pancreatic tumors (79, 80).

The self-renewal and multipotent abilities of early differentiated T cell populations make T memory stem cells and central memory T cells (T_CM_) good candidates for cellular immunotherapy. In this context, IL-21 was shown to exhibit great potential to stimulate CD8^+^ T_SCM_ cells generation and preserve their stem cell-like characteristics. Administration of IL-21-induced CD8^+^ T cells into human melanoma-bearing mice led to the cessation of the tumor growth (19). Furthermore, IL-21 along with other common-γ chain family cytokines, IL-7 and IL-15, have the capacity to expand early-differentiated T cell populations in PMBC-based cultures and the expansion of effector memory T cells in monocyte-derived DCs cultures (81). The presence of IL-21 elevates fatty acid synthesis, mitochondrial biogenesis and antioxidants production, all of these metabolic changes being linked to increased survival and enhanced antitumor efficacy of IL-21-induced CD8^+^ T cells (82). Addition of IL-21 along with IL-15 in the culture media improved the transfection efficiency of human epidermal growth factor receptor-2 (HER-2) chimeric antigen receptor (CAR) T cells and enhanced the cytotoxic activity against HER-2-positive cancer cells (83).

Despite the remarkable success achieved by the adoptive transfer of chimeric antigen receptor T cells in the treatment of hematological malignancies, limited achievements have been made for solid tumor therapy, partly due to the immunosuppressive action of the tumor microenvironment (TME). Nonetheless, genetically modified CAR T to express an IL-4/IL-21 inverted cytokine receptor (4/21 ICR), in which the extracellular domain of the IL-4R is fused to the intracellular domain of the IL-21R, efficiently killed tumor cells producing IL-4 and exhibit an attenuated expression of PD-1 (Programmed cell death protein 1) and Tim-3 (T-cell immunoglobulin domain and mucin domain 3) inhibitory molecules. Also, *in vivo* treatment with 4/21 ICR-CART eradicated established IL-4 positive tumors (84). In another study, CD19-specific CAR T cells engineered to express IL-21 exhibited effector memory phenotypes and enhance antitumor function both *in vitro* and in xenotransplant of B-cell tumors (85).

Batra et al. showed that glypican-3 (GPC3)-CAR T cells that express IL-15, IL-21, or both presented antitumor activity in a model of human hepatocellular carcinoma (HCC). GPC3-CAR T cells that express both IL-15 and IL-21 had a less differentiated profile and continued to proliferate after repeated stimulation with fresh HCC cells. These cells also had a high expression of the gene encoding T-cell factor-1 (TCF-1), a crucial factor involved in the development, expansion, and survival of T cells. GPC3-CAR T co-expressing IL-15 and IL-21 exhibited high expansion and long persistence also *in vivo*, conferring superior antitumor effect and prolonging survival of the mice compared to CAR T cells or CAR T cells expressing a single cytokine. Based on the results from these preclinical studies, two phase I clinical trials enrolling patients with liver tumors were launched and are still ongoing (NCT02932956 and NCT02905188) (86).

### 3.2 NK cells-based adoptive cell therapy

Another category of cells which has therapeutic potential for ACT is NK cells and previous reports have shown IL-21 can be used to expand and improve therapeutic efficacy of CD19 CAR γδT cells or CAR NKT for cancer immunotherapy (87, 88).

NK cells represent a promising therapeutic option due to their unique capacity for spontaneous cytotoxicity against tumor cells and the possibility to use NK cells as a universal donor therapy. NK cells taken from a single donor, after *ex vivo* expansion can be transferred for the treatment of multiple patients because NK cells target tumor cells in an MHC-independent manner and are considered to not induce graft-versus-host disease. Despite having the potential to be a successful therapeutic strategy in the fight against cancer, the use of adoptive NK cell transfer is limited mostly due the requirement of a very large number of NK cells. High doses of NK cells are required for adoptive cell therapy, which can be difficult to obtain from patients, NK cells representing about 5-15% of lymphocytes in the peripheral blood. Various strategies are used for NK cell expansion, but few of these attain highly cytotoxic NK cells for clinical use. To overcome this drawback, *ex vivo* stimulation of NK cells with interleukins, such as IL-2, IL-12, IL-15, IL-18 and IL-21 generate highly cytotoxic NK cells with memory-like behavior.

*Ex vivo* expansion of NK cells in the presence of IL-15 followed by short boost with IL-21 increased the antitumor activity against rhabdomyosarcoma (RMS) cell lines RH30 and RD. The cytotoxic response in this case, is based on elevated degranulation and secretion of pro-inflammatory cytokines like IFN-γ and TNF-α. The adoptive transfer of IL-21 boosted NK cells expansion after radiation therapy induced tumor regression in an RMS xenograft model (89). Also, NK cells generated from CD3/CD19-depleted products stimulated with low dose of IL-15 and a boost with IL-21 short-term before harvesting, exhibited potent cytotoxic activity against human neuroblastoma cell lines SK-N-SH and SK-N-AS (90).

Of note, cytokine stimulation induced highly cytotoxic/activated NK cells, but with limited fold expansions (91). To overcome this shortcoming, autologous and allogeneic cells or “feeder” cells are used to obtain a vigorous expansion and avoid cellular senescence. IL-21 plays an important role in NK cell expansion; many studies reported feeder cells engineered to express membrane-bound IL-21 (mbIL-21) to generate human NK cells with enhanced cytotoxicity, or IL-21 is used to enhance the proliferation of NK cells in the presence of irradiated feeder cells. NK cells cultured in the presence of K562 feeder cells expressing 4-1BBL and mbIL-21 displayed a sustained cell proliferation, over 10 000-fold over 21 days. mbIL-21-expanded NK cells exhibited high cytotoxicity against several human tumor cell lines and produced increased levels of cytokines such as TNF-α, IFN-γ and IL-6. These cells maintained continuous growth over long time and presented an increased telomere length (92).

Adoptive transfer of NK cells expanded with irradiated membrane-bound IL-21/4-1BBL-expressing K562 cells into tumor-bearing mice induced the tumor regression and improved animals survival, indicating a potent therapeutic effect of these NK cells (93). Significantly higher expansion rates of highly cytotoxic NK cells were obtained after *ex vivo* cultivation of NK cells in the presence of autologous feeder cells and medium containing IL-2, IL-15 and IL-21 (94, 95). NK cells from healthy donors expanded in presence of mbIL-21 and 4-1BBL-expressing feeder cells and anti-CD20 monoclonal antibodies, obinutuzumab and rituximab, show enhanced cytotoxic function against chronic lymphocytic leukemia (CLL) cell lines and primary cells through antibody-dependent mechanisms. CLL patient-derived NK cells expanded in the same conditions exhibit increased cytotoxicity against autologous or allogeneic CLL cells. In OSU-CLL and Mec1 cell line xenograft mouse models treatment with expanded NK cells from healthy donors and obinutuzumab and IL-2 significantly increased survival (96).

Culturing of NK cells from healthy donors or multiple myeloma patients with K562 cells genetic engineering to express OX40 ligand and membrane-bound IL-18 and IL-21 improved the rate of NK cell expansion and increase their cytotoxic activity (97). Adding IL-21 only at the beginning of the co-culture of NK cells with irradiated Epstein–Barr virus-transformed lymphoblastoid cell line (EBV-LCL) and IL-2 induced a 53-fold mean NK cell expansion. These expanded NK cells presented an increased antitumor activity in a lung metastases model induced by intravenous injection of SK-MEL-28 cells (98). Ojo et al. developed a novel feeder line named NKF using OCI-AML3 cells, a myeloid leukemia cell line, transduced with mbIL-21. These cells induced vigorous proliferative capacity of NK cells, over 10,000-fold expansion at 5 weeks and the expanded NK cells presented high antitumor activity against mouse models of human leukemia and sarcoma (99).

## 4 Interleukin-21 implementation in clinical trials

Immunotherapy is a curative approach that aims to eradicate tumors by exploiting the features of the immune system. It is used for over 100 years and includes cytokines, adoptive cell transfer therapy, tumor‐specific vaccines, immune checkpoint inhibitors, and immunomodulatory drugs. Based on its characteristics observed in preclinical studies, IL-21 is currently used in clinical trials for cancer immunotherapy, as a single agent or in combination with other drugs, as summarized in **Table 1**. Additionally, IL-21 is used to modulate the antitumor properties of immune cells in adoptive cell therapy (**Table 2**).

**Table 1:** Clinical trials of interleukin-21 in cancer immunotherapy.

Clinical Trial	Phase	Start date/Status	Condition or disease	Description	Responsible Party/Location	Publications associated to the study
NCT00095108	1	2004 Completed	Stage IV, malignant melanoma or kidney cancer	Safety and efficacy study of i.v. rIL-21	ZymoGenetics, United States	(100–102)
NCT00336986	2	2004 Completed	Metastatic Melanoma	Efficacy study of i.v. rIL-21	Novo Nordisk A/S, Australia	(103, 104)
NCT00347971	1	2006 Completed	non-Hodgkin’s lymphoma	Safety study of i.v. rIL-21 in combination with anti-CD20 mAb rituximab (rituxan)	ZymoGenetics, United States	(105)
NCT00389285	1/2	2006 Completed	Metastatic renal cell carcinoma	Safety study of i.v. rIL-21 in combination with VEGFR tyrosine kinase inhibitor sorafenib	ZymoGenetics, United States	(106)
NCT00523380	2	2007 Completed	Ovarian Cancer	Efficacy study of i.v. rIL-21 (NN028) in combination with Caelyx (pegylated liposomal doxorubicin)	Novo Nordisk A/S, France and Germany	No publications available
NCT00514085	2	2007 Completed	Metastatic or recurrent malignant melanoma	Safety and efficacy study of i.v. rIL-21	Canadian Cancer Trials Group, Canada	(107)
NCT00617253	2	2008 Completed	Stage IV Renal Cell Carcinoma	Dose escalation safety study of s.c. rIL-21 (NN028) in combination with multitargeted tyrosine kinase inhibitor sunitinib (Sutent)	Novo Nordisk A/S, Germany and Netherlands	(108)
NCT00601861	2	2008 Discontinued not due to any safety concerns	Stage III melanoma	Comparison study of the pathology in lymph nodes before and after the effect of s.c. rIL-21	Novo Nordisk A/S, Germany	No publications available
NCT01152788	2	2010 Completed	Metastatic or recurrent melanoma	Comparative study of i.v. rIL-21 versus dacarbazine	Canadian Cancer Trials Group, canada and United States	(109)
NCT01489059	1	2011 Completed	Melanoma	Safety and clinical benefits study of i.v. rIL-21(BMS-982470) and anti-CTLA-4 antibody Ipilimumab (Yervoy)	Bristol-Myers Squibb, United States and Puerto Rico	No publications available
NCT01629758	1	2012 Completed	Advanced or Metastatic Solid Tumors	Safety and clinical benefits study of i.v. rIL-21(BMS-982470) and anti-PD-1 antibody Nivolumab (BMS-936558)	Bristol-Myers Squibb, United States	No publications available
NCT04362748	1	2020 Recruiting	Advanced Solid Tumor	Safety, tolerability and maximum tolerated dose study of i.v. bifunctional fusion protein comprising a PD-1-targeting antibody and IL-21 mutein (AMG 256, Latikafusp)	Amgen, United States, Australia, Belgium, Spain	No publications available
NCT05296772	1	2022 Recruiting	Advanced cancer or lymphoma	Safety and efficacy study of i.v. fusion protein JS014 (Interleukin 21 and humanized anti-human serum albumin VHH antibody) alone or in combination with anti-PD-1 antibody Pembrolizumab (Keytruda)	Anwita Biosciences, Taiwan	No publications available
NCT05914376	1	2023 Recruiting	Advanced Solid Tumors	Safety, tolerance, pharmacokinetics, and biological properties of recombinant human IL-21 oncolytic vaccinia virus injection (hV01) intratumoral administration.	Hangzhou Converd Co., Ltd., China	No publications available

rIL-21, recombinant interleukin-21; i.v., intravenous; VEGFR, vascular endothelial growth factor receptor; s.c., subcutaneous; CTLA-4, Cytotoxic T-lymphocyte associated protein 4; PD-1, Programmed cell death protein 1.

**Table 2:** Clinical trials of interleukin-21 use for adoptive cell therapy in cancer immunotherapy.

Clinical Trial	Phase	Start date/Status	Condition or disease	Drug	Description	Responsible Party/Location	Publications associated to the study
NCT00823524	1/2	2009 Completed	Hematologic malignancies or solid tumors	NK cells cultivated in media with IL-15, IL-21 and hydrocortisone	Side effects and best dose study of donor-derived NK cells after HLA-haploidentical hematopoietic cell transplantation	Asan Medical Center, Republic of Korea	No publications available
NCT01087294	1	2010 Active, not recruiting	Recurrent or persistent B-cell malignancies after allogeneic stem cell transplantation	Anti-CD19-CAR-transduced T cells cultured in media with IL-21, IL-7, and glycogen synthase kinase-3β inhibitor TWS119	Safety and effectiveness study of administering allogeneic anti-CD19-CAR-transduced T cells	National Cancer Institute (NCI), United State	(110)
NCT01106235	1	2010 Terminated	Stage IV Melanoma	Autologous IL-21 modulated CTLs	Side effects and best dose study of CTLs together with cyclophosphamide and rIL-2 (aldesleukin)	Fred Hutchinson Cancer Research Center, United States	No publications available
NCT02809092	1/2	2017 Unknown	Acute Myeloid Leukemia	mbIL-21-Expanded Haploidentical NK Cells	Safety and feasibility of IL-21-expanded NK cells after chemotherapy with Fludarabine, Cytarabine, and G-CSF	Hospital de Clinicas de Porto Alegre, Brazil	No publications available
NCT02932956	1	2018 Active, not recruiting	Liver Cancer	GAP T cells (GPC3-CAR T cells expressing IL-15, IL-21 and 4-1BB costimulatory endodomain-”GBBz”)	Safety and dosage study of GAP T cells along with lymphodepleting chemotherapy (Cyclophosphamide and Fludarabine)	Baylor College of Medicine, United States	No publications available
NCT03348033	1/2	2019 Unknown	Chronic Myeloid Leukemia	Autologous NK Cells expanded on clone 9 K562 aAPC expressing mIL-21	Safety and feasibility study of NK cells expanded on mIL-21-aAPC after chemotherapeutic conditioning with Fludarabine, Cytarabine, and G-CSF	Federal University of Rio Grande do Sul, Brazil	No publications available
NCT02905188	1	2019 Completed	Hepatocellular Carcinoma	GLYCAR T cells (GPC3-CAR T cells expressing IL-15, IL-21 and 4-1BB costimulatory endodomain-”GBBz”)	Safety and dosage study of GLYCAR T cells along with lymphodepleting chemotherapy (Cyclophosphamide and Fludarabine)	Baylor College of Medicine, United States	No publications available
NCT04729543	1/2	2020 Recruiting	Melanoma, Head and Neck Cancer	Autologous MAGE-C2/HLA-A2 TCR T cells (MC2 TCR T cells), young T cells generated by IL-15 and IL-21 stimulation	Safety and efficacy study of MC2 TCR T cells combined with epigenetics drugs (5’ Azacytidine and Valproic acid) and IL-2	Erasmus Medical Center, Netherlands	(111–114)
NCT04093648	1	2020 Withdrawn (the key elements of the study were incorporated into another study)	Hepatocellular Carcinoma, Hepatoblastoma	TEGAR T cells (GPC3-CAR T cells expressing IL-15 and IL-21)	Safety and dosage study of TEGAR T cells along with lymphodepleting chemotherapy (Cyclophosphamide and Fludarabine)	Baylor College of Medicine, United States	No publications available
NCT04220684	1	2020 Recruiting	Recurrent/refractory acute myeloid leukemia	mbIL-21-expanded, off-the-shelf, third-party donor-derived NK cells	Safety study of mbIL-21-expanded NK cells after chemotherapy with Fludarabine and Cytarabine	Ohio State University Comprehensive Cancer Center, United States	No publications available
NCT05503134	1/2	2022 Recruiting	Relapsed or refractory acute myeloid leukemia	Universal donor mbIL-21 expanded NK cells (UD-NK), Fludarabine, Cytarabine	Dose escalation, safety and efficacy study of UD-NK cells after chemotherapy with Fludarabine and Cytarabine	Nationwide Children’s Hospital, United States	No publications available
NCT04848064	1	2022 Recruiting	Relapsed/refractory cutaneous T-cell lymphoma and adult T-cell leukemia/lymphoma	IL-21 expanded, off the shelf, third-party NK cells	Safety, tolerability, and MTD study of NK cells in combination with anti-CCR4 antibody Mogamulizumab after chemotherapy with Fludarabine and Cytarabine	Ohio State University Comprehensive Cancer Center, United States	No publications available
NCT04715191	1	2024 Not yet recruiting	Pediatric solid tumors (Liver Cancer, Rhabdomyosarcoma, Liposarcoma, Malignant Rhabdoid Tumor, Wilms Tumor Yolk Sac Tumor)	CARE T cells (GPC3-CAR T cells expressing IL-15 and IL-21)	Safety and dosage study of CARE T cells along with lymphodepleting chemotherapy (Cyclophosphamide and Fludarabine)	Baylor College of Medicine, United States	No publications available

IL-21, interleukin-21; NK, Natural Killer; IL-15, interleukin-15; HLA, Human leukocyte antigen; CAR, Chimeric Antigen Receptor; IL-7, interleukin-7; CTLs, Cytotoxic T lymphocytes; rIL-2, recombinant interleukin-2; mbIL-21, membrane-bound IL-21; G-CSF, Granulocyte colony-stimulating factor; aAPC, artificial Antigen Presenting Cells; TCR, T cell receptor; CCR4, chemokine (C-C motif) receptor-4.

The first clinical trials using recombinant interleukin-21 (rIL-21) started in 2004 and were conducted in patients with Melanoma or Renal Cell Carcinoma. A phase I study conducted in the United States enrolled 24 patients with Metastatic Melanoma (MM) and 19 patients with Metastatic Renal Cell Carcinoma (RCC) (NCT00095108). Patients were treated with two five-day cycles intravenous injections of rIL-21, every cycle followed by a rest period. In patients with MM the treatment with rIL-21 was associated with 1 complete response (CR), 11 stable disease (SD), and 12 progressive diseases (PD), while in patients with RCC was observed 4 with partial response (PR), 13 with SD, and two with PD. The rIL-21 therapy at 30 μg/kg was well tolerated with evidence of antitumor activity and without evidence of vascular leak syndrome. The common adverse events were flu-like symptoms, pruritus, and rash. Patients presented adverse effects mild to moderate in severity, such as abdominal pain, cytopenias, hypophosphatemia, and increased hepatic enzymes. The dose recommended for further study was 30 µg per kg (100).

Another study was conducted in Australia on patients with Stage IV Melanoma (NCT00336986). In phase I of the study, the safety and tolerability of rIL-21 at various dose were assessed on 29 patients. The rIL-21 treatment was administrated thrice weekly for 6 weeks (3/wk) or three five-day cycles with a rest period between cycles. Similar adverse effects were reported and the same maximum tolerated dose (30 μg/kg) for both treatment regimens as for the previous study. After treatment from phase I, one partial response and nine stable diseases were observed. In phase II of the study, the effect of rIL-21 on tumor size was assessed. The chosen dosing regimen was 30 μg/kg of rIL-21 for five-day cycle followed by nine days rest for 6 weeks. The overall response rate for the study was 8.3% with one confirmed complete response. rIL-21 therapy induced dose-dependent increases in soluble CD25 and up-regulated mRNA expression levels of IFN-γ, perforin and granzyme B in CD8+ T and NK cells (101–103).

Schmidt et al. reported that IL-21 administrated subcutaneously 3 days per week for 8 or 16 weeks, revealed antitumor activity in patients with MM or RCC. Subjects experimented adverse effects like those found in the other studies and the dose of 200 μg/kg was chosen to be the maximum tolerated dose. The levels of soluble CD25 increased in a dose-dependent manner, and higher doses induced mRNA expression of the IFN-γ, perforin, and granzyme B in peripheral blood mononuclear cells. The overall disease control rate was 69%; three patients, one MM and two RCC, with the overall best tumor response (115).

Another phase II study assessed the efficacy of IL-21 treatment in patients with metastatic or recurrent malignant melanoma (NCT00514085). Treatment with IL-21 showed promising antitumor activity, the overall response rate being 22.5% and the median overall survival being 12.4 months (107). Based on these results, the same group evaluating the efficacy of rIL-21 versus chemotherapy with dacarbazine in patients with MM (NCT01152788) showed that the treatment with IL-21 was comparable to dacarbazine (109). A study that aimed to evaluate the effect of IL-21 on metastases in lymph nodes in patients with stage III melanoma was discontinued due the decision of sponsoring company (NCT00601861).

All this data showed that rIL-21 has modest but consistent clinical activity in melanoma and RCC as a single agent. Consequently, clinical studies of IL-21 combined with therapeutic mAbs or tyrosine kinase inhibitors were designed.

The safety, tolerability and efficacy of rIL-21 in combination with rituximab (anti-CD20 monoclonal antibody) were assessed in patients with B-Cell Non-Hodgkin’s Lymphoma (NCT00347971). The standard rituximab therapy in combination with 100 µg/mL rIL-21 was well tolerated, the most common adverse effects similar to the ones reported in previous trials of rIL-21 in MM and RCC. Positive responses were observed in 42% of patients with indolent B-cell malignancies, with durable remissions in four patients (105). In a phase I/II study the combination of rIL-21 with sorafenib, a vascular endothelial growth factor receptor (VEGFR) tyrosine kinase inhibitor was investigated in patients with metastatic renal carcinoma (NCT00389285). In phase I, the IL-21 maximum tolerated dose was established at 30 µg/mL along with sorafenib at the standard dose. This combination had antitumor activity with the majority of patients showing tumor reduction and 21% of patients meeting objective response criteria (106).

A study that explored the safety and tolerability of rIL-21 administered subcutaneously in combination with sunitinib (an inhibitor of VEGFR, Platelet-derived growth factor receptor, c-KIT, and FLT-3) was conducted in patients with metastatic renal cell carcinoma (NCT00617253). The dose of 10 μg/kg IL-21 and sunitinib induced hematologic dose-limiting toxicities, while the dose level of 3 μg/kg rIL-21 had no clinically relevant effects. The study was discontinued due to adverse effects (108). A phase I study assessed effects of rIL-21 plus cetuximab, an epidermal growth factor receptor (EGFR) inhibitor, in patients with metastatic colorectal cancer who had not received chemotherapy. The study was closed without establishing the maximum tolerated dose. The treatment was well tolerated and preliminary data sustained the feasibility of therapy IL-21 in combination with cetuximab (116).

The antitumoral potential of IL-21 in combination with chemotherapy or immune checkpoint blockers is currently evaluated in several clinical studies, but the outcome is not yet available. The therapeutic efficacy of the combination of IL-21 with a chemotherapeutic agent (pegylated liposomal doxorubicin, Caelyx) is investigated for ovarian cancer patients (NCT00523380), while the safety and tolerability of rIL-21 in combination with the anti-CTLA4 mAb (Ipilimumab) or the anti-PD-1 mAb (Nivolumab) are evaluated in patients with unresectable stage III/IV melanoma (NCT01489059) or advanced or metastatic solid tumors (NCT01629758).

A phase I study using a Bayesian Optimal Interval (BOIN) design was recently started to assess the safety and efficacy of a recombinant fusion protein of IL-21 and humanized anti-human serum albumin VHH antibody (JS014) in patients with advanced cancer (NCT05296772). The study includes two parts, the first part to test JS014 as a single agent, and the second to evaluate JS014 in combination with anti-PD-1 mAb (pembrolizumab).

Oncolytic viruses represent a promising approach in cancer therapy too as highlighted by the results obtained in pre-clinical studies and the first oncolytic virus T-VEC (Imlygic) for cancer immunotherapy was approved by the U.S. Food and Drug Administration (FDA) in 2015. Based on preclinical studies, which indicated potent antitumor activity of oncolytic viruses armed with IL-21, a clinical study was started in 2023 and aims to evaluate the safety of recombinant human IL-21 oncolytic vaccinia virus injection (hV01) in patients with advanced solid tumors (NCT05914376). This novel oncolytic virus hV01 was engineered by the deletion of the viral growth factor and viral thymidine kinase genes and armed by with human IL-21 gene.

On the other side, the number of clinical studies involving ACT is rapidly increasing, and new ways sought to improve the curative effects and limit the side effects of this approach are uncovered. Another potential clinical application of IL-21 in cancer therapy is its use generating potent cytotoxic cells such as CD8^+^ T cells and NK cells for adoptive cell therapy (117, 118). For example, treatment with haploidentical NK cells generated from CD3-depleted PBMC and cultivated in media supplemented with IL-15, IL-21 and hydrocortisone was well tolerated, without acute side effects, and provided a reduction in leukemia progression, especially in patients with acute myelogenous leukemia (49) (NCT00823524).

Although chimeric antigen receptor T-cell therapy produced important clinical responses in some blood cancers, there are major limitations to this therapy which still must be addressed, and IL-21 could be used to improve clinical efficacy of this therapeutic approach. In several clinical trials, IL-21 is added in the culture media to potentiate the antitumor effects of CAR T cells. The allogeneic T cells expressing a chimeric antigen receptor targeting the B-cell antigen CD19 and cultured in media containing IL-21 are used for treatment of patients with recurrent or persistent B-cell malignancies (110) (NCT01087294). Also, IL-21 modulated melanoma antigen recognized by T cell (MART)-1 specific CD8+ T cells which were used in treating patients with metastatic melanoma following cyclophosphamide conditioning (NCT01106235). In a phase I/II study patients with melanoma-associated antigen-encoding (MAGE)-C2-positive melanoma and Head and Neck Cancer will be treated with T cell receptor Gene-engineered T cells. IL-21 and IL-15 will be used to generate T cells with young phenotype (NCT04729543). Similarly, based on previous preclinical data, three clinical trials will test GPC3-CAR T cells co-expressing IL-15 and IL-21 in patients with GPC3-positive solid tumors (86, 119) (NCT02932956, NCT02905188, NCT04715191).

Feeder cells engineered to express membrane-bound IL-21 allow longer cultivation of NK cells and several clinical trials using mbIL-21-expanded NK cells have been started recently. The purpose of these studies is to determine the safety, feasibility, and maximum tolerated dose of mbIL-21-expanded NK cells after fludarabine/cytarabine chemotherapy in myeloid malignancies (NCT01787474, NCT02809092, NCT03348033, NCT04220684, NCT05503134). mbIL-21–expanded NK cells treatment in patients with high-risk myeloid malignancies did not show major safety concerns without dose-limiting toxicities or increased risk of graft-versus-host disease (GVHD) (120–122) (NCT01787474, NCT02809092). Also, a phase I trial was designed to assess the effects of mbIL-21-expanded NK cells in combination with mAb targeting CC chemokine receptor 4 mogamulizumab in patients with relapsed/refractory cutaneous T-cell lymphoma and adult T-cell leukemia/lymphoma (NCT04848064).

## 5 Conclusions and perspectives

Interleukin-21 is a potent 4α-helix bundle type 1 cytokine that showed promising impact on cancer immunotherapy by augmenting the antitumor responses of immune cells and supporting the differentiation of memory T cells. Although it was discovered fairly recently, it’s role in therapy has been extensively investigated. If early studies focused on the administration of IL-21 as monotherapy, recent studies have investigated optimal methods of delivering IL-21 or combining it with different drugs to increase its therapeutic potential and reduce adverse effects. Clinical studies have demonstrated the efficacy and safety of combined therapies, the treatment with IL-21 alone was associated mainly with disease stabilization in some melanoma and renal cancer patients (17, 100).

Treatment with IL-21 did not lead to the accumulation of a sufficiently number of tumor-infiltrating lymphocytes, either due to rapid consumption in the periphery or the short half-time. To address this limitation, various bifunctional fusion proteins have been obtained by binding IL-21 to tumor-targeting antibodies; these products exhibited increased stability and prolonged half-life and allowing IL-21 to be delivered directly to tumor infiltrating lymphocytes. Constructs generated by fusion IL-21 to anti-CD20, anti-EGFR or monoclonal antibody that target Claudin18.2 showed extended half-life and enhanced antitumor efficacy *in vitro* and *in vivo* studies (59, 65). IL-21 mutein fused to anti-PD-1 antibody improved CD8^+^ T cell function and antitumor response in a humanized mouse model. Based on these encouraging results a phase I clinical trial was designed to evaluate safety, tolerability, and estimated dosing of the anti-PD-1/IL-21 fusion protein in patients with advanced solid tumors (62, 63).

IL-21 can also achieve increased potency by combination with other therapies such as monoclonal antibodies targeting proteins on the cancer cells (61, 67, 69), tyrosine kinase inhibitor drugs (105, 106, 108, 116), immune checkpoint inhibitors (66, 69, 70), or other cytokines (24, 89, 90). Oncolytic viruses destroy the tumor cells mainly by direct lysis and indirect induction of both innate and adaptive immune responses. Oncolytic viruses expressing IL-21 elicit potent antitumor activity as monotherapy or in combination with other immunotherapies (CAR T or iNKT cells, immune checkpoint inhibitor and PI3Kδ inhibitor) (71–75).

As we described above, interleukin-21 is a multifunctional cytokine which regulates a wide range of immune cells, especially the cytotoxic lymphocytes, promoting the antitumor response. Many encouraging pre-clinical and clinical data indicate IL-21 as a potent agent for cancer immunotherapy. However, it was shown that IL-21 is implicated in the pathogenesis of numerous inflammatory and autoimmune diseases such as: inflammatory bowel diseases, Bechet’s disease, psoriasis, rheumatoid arthritis, juvenile idiopathic arthritis, type I diabetes, systemic lupus erythematosus, multiple sclerosis, pemphigus, thrombocytopenia, Sjogren’s syndrome, vitiligo, autoimmune thyroid disease, and graft-vs. host disease; the elevated levels of IL-21 or IL-21 mRNA being detected in patients with these pathologies (123). There are also evidences showing production in excess of IL-21 in the mucosa of patients with ulcerative colitis and in the neoplastic areas of patients or mice with colitis-associated colorectal cancer and sporadic colorectal cancer (124–126). All these data indicated a pro-tumorigenic activity of IL-21 which is associated with the promotion of T_h_7 phenotype. If in colitis-associated colorectal cancer IL-21 appears to have a pro-tumorigenic role, in sporadic intestinal carcinogenesis IL-21 impairs tumor growth and deficiency of this cytokine is associated with enhancement of the T_h_17 differentiation (127). Further investigations are needed to explore the exact role of IL-21 in the T_h_17 pathway and its implication in tumorigeneses.

It was shown that IL-21 modulate B cell proliferation and can acts as a stimulator factor for terminally differentiated B-cell malignancy such as multiple myeloma or Epstein-Barr virus-positive diffuse large B cell lymphoma (128–130). In hematological malignancies depending on the neoplastic cell type IL-21 can have pro-tumorigenic activity or antitumorigenic properties inducing apoptosis in malignancies such as: chronic lymphocytic leukemia, follicular lymphoma, diffuse large B cell lymphoma, and Mantle cell lymphoma (13).

Thus, the combined effects of IL-21 with anti-CD20 mAb, anti-PD-1 antibody or other tumour-targeted antibodies which mediate the antibody-dependent cellular cytotoxicity and generation of tumour-specific T cells can enhance the therapeutic effects. Future studies are required to shed light on the pleotropic molecular pathways of IL-21 signaling and their implications in cancer therapy.

The antitumor potential of IL-21 was also exploited for the preparation of immune cells for adoptive transfer. Incorporating IL-21 into CAR T, NKT or γδT cells potentiated cell persistence and significantly enhanced antitumor effects *in vitro* and *in vivo* models. IL-21 in combination with other cytokines are used to optimal expanded TILs, CAR T cells and NK cells prior to their use in adoptive cell therapy. IL-21 expanded cells exhibited enhanced antitumor activity upon adoptive transfer. Additionally, IL-21-expressing antigen-presenting cells represent an important tool for the generation of NK cells and tumor-reactive CD8^+^ T cells with powerful antitumor effects and self-renewal capacity.

As combined therapy has become common practice in cancer treatment the number of studies aiming to develop better combinations and safety evaluation studies to improve this therapeutic approach has exponentially increased. In this context, the use of IL-21 in combination with either chemotherapy or immunotherapy agents holds great potential with numerous combinations already tested in preclinical studies (see **Figure 3**) as well as some clinical trials (see **Table 1**). Therefore, in our perspective combinatorial therapy including IL-21 is the most promising and realistic perspective to cancer therapy.

Another breakthrough of cancer immunotherapy, with excellent results for treating certain cancer types, is ACT. This has shown excellent results for hematological malignancies, with very little side effects, and currently sustained efforts are being made to design ACT which overcomes the TME inhibitory effect for solid tumors. T cell-mediated ACT has been the most used therapy with very good results both in preclinical and clinical trials, however recent data suggests this therapeutic is under investigation for potential causing lymphomas or leukemia (131). Therefore, this will require a reevaluation of the potential side effects of this therapy and a thorough assessment is compulsory. Alternatively, ACT for cancer therapy could be designed using CAR NK cells, a less exploited curative approach but which could potentially compensate for the side effects of CAR T-mediated therapy. This is still unexploited and harnessing NK potential for designing cancer treatments with minimal side effects is an asset.

In conclusion, significant amount of research on IL-21 reveals its critical role in the activation of immune system and its antitumor potential. The best IL-21-based constructs are currently being developed for the treatment of cancer and some of these are in early phase clinical trials. Although limited results of using IL-21 in cancer therapy have been published so far, the various way of using of IL-21 and the increasing number of registered clinical trials reflects the considerable interest in IL-21 for treatment of cancer.

## Author contributions

GI: Writing – original draft, Writing – review & editing, Conceptualization. MC: Writing – original draft, Writing – review & editing.
